# Alternative promoters of *Peg3* with maternal specificity

**DOI:** 10.1038/srep24438

**Published:** 2016-04-14

**Authors:** Bambarendage P. U. Perera, Joomyeong Kim

**Affiliations:** 1 Department of Biological Sciences, Louisiana State University, Baton Rouge, LA 70803, USA

## Abstract

*Peg3* (paternally expressed gene 3) is an imprinted gene localized within an evolutionarily conserved 500-kb domain in human chromosome 19q13.4 and mouse proximal chromosome 7. In the current study, we have identified three alternative promoters for mouse *Peg3* and one alternative promoter for human *PEG3*. These alternative promoters are localized within the 200-kb upstream region of human and mouse *PEG3*, which is well conserved and thus predicted to harbor several *cis*-regulatory elements for the *PEG3* domain. In the mouse, two of these alternative promoters drive maternal-specific expression of *Peg3* specifically in the hypothalamus of the adult brain, while the remaining third promoter drives bi-allelic expression of *Peg3* with a paternal bias only in the neonatal-stage brain. In human, an alternative transcript is also detected at relatively very low levels in adult brain and placenta. Overall, the identification of alternative promoters in both mouse and human models suggests that these alternative promoters may be functionally selected features for the *PEG3* imprinted domain during mammalian evolution.

*Peg3* (paternally expressed gene 3) is the first imprinted gene identified from an evolutionarily conserved 500-kb domain localized in human chromosome 19q13.4 and mouse proximal chromosome 7^1^,^2^,^3^. This domain harbors 6 additional imprinted genes: the paternally expressed *Usp29*, *Zfp264*, and *APeg3*, and the maternally expressed *Zim1*, *Zim2*, and *Zim3*^4^,^5^,^6^,^7^. *Peg3* is structurally comprised of 9 exons that are spread over a 25-kb distance in both human and mouse. Interestingly, the 200-kb upstream region of both human and mouse *PEG3* lacks any obvious ORF (Open Reading Frame), but this region has been well preserved during mammalian evolution. According to recent studies, this 200-kb region is filled with small ECRs (Evolutionarily Conserved Regions), which are putative *cis*-regulatory elements for *Peg3* and other imprinted genes^8^,^9^. *Peg3* has been shown to be involved in controlling maternal-caring behaviors and fetal growth rates^10^. Accordingly, *Peg3* is highly expressed in neuronal cells as well as embryos and placentas^3^,^11^. The mechanistic basis for these expression patterns and also the paternal-specific expression of *Peg3* is still under investigation. Nevertheless, the potential *cis*-regulatory elements found within the 200-kb region are hypothesized to be involved in regulating the tissue- and allele-specific expression patterns of *Peg3*, given their unusual evolutionary conservation.

As seen in the *Peg3* locus, other imprinted domains also have similar unusual genomic layouts. For instance, the upstream region of human and mouse *SNRPN* is more than 500 kb in length, which is relatively large compared to a 20-kb transcribed area from its coding region. This 500-kb region also lacks any obvious ORFs, but this region harbors multiple alternative promoters for *Snrpn* and *Ube3a-ATS*, an antisense transcript gene to *Ube3a*^12^. In fact, some of these alternative promoters are critical for establishing germline-specific DNA methylation marks^13^,^14^. A similar case has been observed from the *Gnas* domain, where the transcription of the locus is driven by multiple alternative promoters of various tissue and allele specificity^15^,^16^. Yet, the transcription by one particular promoter during oogenesis is believed to be responsible for establishing oocyte-specific DNA methylation marks for the downstream promoter^17^. There are additional cases of imprinted domains that harbor multiple alternative promoters in their large upstream regions, including *Zac1* and *Grb10*. In the *Zac1* locus, the multiple promoters are responsible for its various tissue-specific expression patterns^17^,^18^,^19^. On the other hand, the two alternative promoters of *Grb10* are known to display both allele and tissue specificity^20^,^21^. Given these examples, it is reasonable to predict that the 200-kb upstream region of the *Peg3* locus may also have similar alternative promoters, although the *Peg3* locus has long been known to have only one promoter in both human and mouse.

A recent study, however, demonstrated that the expression of *Peg3* appears to be bi-allelic in certain areas of the adult mouse brain, such as the hypothalamus and choroid plexus^22^. This may be an indicator for either de-repression of the maternal allele of the known promoter or the presence of potential alternative promoters for the *Peg3* locus. In the current study, therefore, we sought to characterize the observed bi-allelic expression pattern with a strategy involving 5′ RACE and NGS-based (Next Generation Sequencing) deep sequencing experiments. With this approach, we were able to identify several alternative promoters for both human and mouse *PEG3*. A series of expression analyses further revealed that these alternative promoters display allele-, tissue-, and stage-specific expression patterns. More detailed results are described in the following section.

## Results

### Mutant alleles for *Peg3*

**Figure 1:**
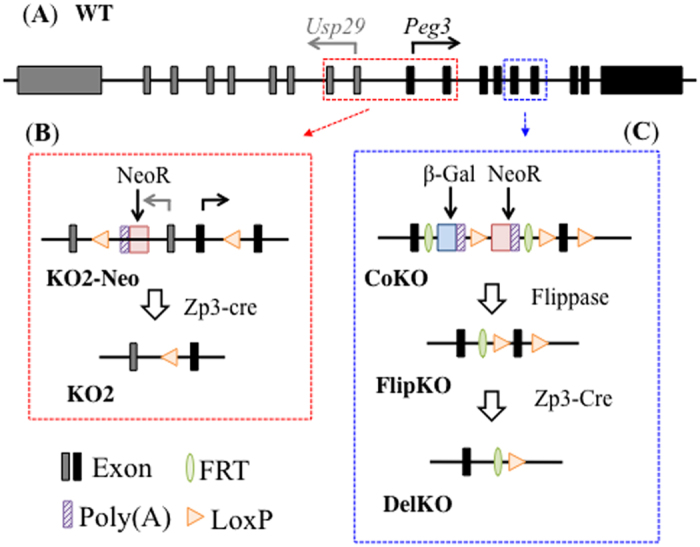
Genomic structures of the wild-type and mutant alleles of *Peg3*. (**A**) Schematic representation of the wild-type alleles of mouse *Usp29* and *Peg3* loci. Gray and black boxes indicate the exons of *Usp29* and *Peg3*, respectively. Transcriptional direction for each gene is represented by arrows with corresponding colors. (**B**) Schematic representation of the KO2 mutant allele. The KO2-Neo mutant has a 6.0-kb insertion containing a PGK promoter-driven neomycin resistance gene (*NeoR*) as selection marker, shown by the red box. *NeoR*, along with the 4.0-kb promoter region of *Peg3* containing first exons of *Usp29* and *Peg3* are flanked by two LoxP sites as indicated with triangles. Cre-recombinase was used to delete the first exons of *Usp29* and *Peg3*, producing the KO2 mutant. (**C**) Schematic representation of the DelKO mutant allele. The conditional-ready knockout (CoKO) allele has a 7.1-kb insertion containing a promoterless β-galactosidase gene (*β-Gal*) as shown by the blue box and human β-actin promoter-driven neomycin resistance gene (*NeoR*) as shown by the red box. The insertion cassette of the CoKO allele is removed by FLP-mediated recombination, producing the FlipKO allele which has the 6^th^ exon of *Peg3* flanked by two LoxP sites as indicated with triangles. Cre recombinase was used to delete exon 6, producing the DelKO allele for the *Peg3* locus.

In the current study, two mutant alleles of mouse *Peg3* were used to identify the alternative 1^st^ exons and corresponding promoters that may be localized upstream of the known 1^st^ exon and promoter of *Peg3*. The known 1^st^ exon of *Peg3* is localized next to the 1^st^ exon of the adjacent gene *Usp29*, controlling the transcription of both *Peg3* and *Usp29* bidirectionally (Fig. 1A). The 4-kb genomic region surrounding this bidirectional promoter is also differentially methylated between two alleles: unmethylated paternal versus methylated maternal alleles^8^. Thus, this promoter is functional only from the paternal allele. This 4-kb DMR of *Peg3* was deleted using a floxed allele recently generated in the lab named KO2-Neo (H. He *et al*., unpublished). The Zp3-cre strain involves a cre-recombinase enzyme that is expressed in the female germ line. When bred with the KO2-Neo strain, the floxed sequence is recognized and deleted in the growing oocytes. Thus, generating the KO2 allele with a deletion of the known *Peg3* promoter region including its first exon (Fig. 1B,C). The current study also used another strain, CoKO, which is designed to truncate the transcription of *Peg3* and also to delete the exon 6 with two flanking LoxP sites^23^. This CoKO strain was first bred with the Flippase strain, and later with the Zp3-cre strain to delete exon 6, generating the second mutant allele DelKO for the current study (Fig. 1C). Since *Peg3* is expressed mainly from the paternal allele, the two mutant alleles were transmitted paternally to remove the main transcript of *Peg3* for the KO2 strain and also to have the main transcript without exon 6 for the DelKO strain^24^.

### Alternative 1^st^ exons and promoters of *Peg3*

**Figure 2:**
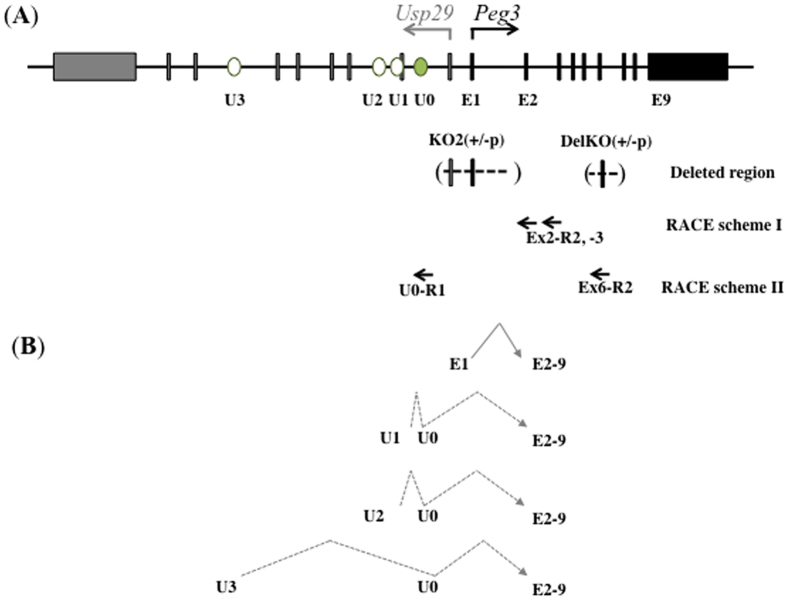
Alternative 1^st^ exons and promoters of *Peg3*. (**A**) Map of the mouse *Peg3* locus. Gray and black boxes indicate the exons of *Usp29* and *Peg3*, respectively. Transcriptional direction for each gene is represented by arrows with corresponding colors. The solid boxes indicate the position of the exons of *Peg3*, labeled E1 through E9, followed by ovals to indicate the position of upstream alternative exons. The closed oval represents the shared upstream exon U0, and open ovals represent alternative 1^st^ exons U1, U2, and U3, respectively. The deleted *Peg3* exons corresponding to KO2^(+/−p)^ and DelKO^(+/−p)^ mutant alleles are shown using parentheses and dashed lines. The two extended arrows show the anchoring primers used for nested PCR after 5′ RACE: Ex2-R2 and Ex2-R3 for KO2^(+/−p)^ mutant and its WT counterpart, as shown by scheme I. The two extended arrows underneath it shows the anchoring primers used for nested PCR after 5′ RACE: Ex6-R3 and U0 for DelKO^(+/−p)^ adult hypothalamus and a WT neonate brain as indicated by the scheme II. A summary of the sequence reads is shown in Table 1. (B) The exon structures of *Peg3* alternative transcripts identified from the mouse brain. The E1 - E2 exon structure indicates the transcription by the main promoter of *Peg3*. The exon U1 is positioned 20-kb upstream of E1, the 1^st^ exon of *Peg3*. The alternative transcript starting from the U1 exon connects to U0 (16-kb upstream of E1) and E2 exons while skipping E1. The exon U2 is positioned 26-kb upstream of E1. This alternative transcript starting from the U2 exon connects to U0 and E2 exons while skipping U1 and E1 exons. The exon U3 is positioned 163-kb upstream of E1. The alternative transcript starting from the U3 exon connects to U0 and E2 exons while skipping U2, U1 and E1 exons. Genomic distances are not drawn to scale.

To identify potential alternative 1^st^ exons of *Peg3*, a series of 5′ RACE (Rapid Amplification cDNA Ends) experiments were performed using total RNA isolated from the tissues of the two mutant strains and their wild-type littermates (Fig. 2A). Total RNA was isolated from the brains of neonates given that high levels of *Peg3* expression were found in neuronal cells^3^. The hypothalamus of adult mice has already shown maternal expression of *Peg3*, suggesting the presence of potential alternative promoters for *Peg3*^22^. Thus, this portion of brain was also included for the current study. The total RNA from the neonatal brains and the adult hypothalamus were first reverse-transcribed with gene-specific primers: Ex2-R1 for the total RNA from KO2^(+/−p)^ and Ex6-R1 for the total RNA from DelKO^(+/−p)^. These initial cDNA fragments generated by Ex2-R1 and Ex6-R1 primers were further amplified using two different nested PCR schemes (RACE I and II, Fig. 2A). The amplified libraries were finally sequenced using a NGS (Next Generation Sequencing) platform^25^, and the results are summarized in Table 1.

Inspection of the sequence reads from four individual libraries provided the following conclusions (Table 1). First, detailed analyses confirmed the presence of four new upstream exons, thus named U0 through U3. The genomic positions of these exons relative to that of the known 1^st^ exon (E1) are as follows: U3 (163 kb), U2 (26 kb), U1 (20 kb) and U0 (16 kb upstream of E1). These upstream exons are spread throughout the middle 200-kb genomic region of the *Peg3* imprinted domain, which is quite unexpected and remarkable, as the previously identified exons of *Peg3*, E1-E9, spans only a 25-kb genomic distance (Fig. 2B). Second, the three exons U1, U2, and U3 have a clear exon-intron border at their 3′-ends, but not at the 5′-ends, suggesting that the 5′-end of these exons likely contain transcription start sites. This further suggests that the immediate upstream regions of these three exons should serve as alternative promoters for the *Peg3* locus. This is also the case for E1 exon, which has long been known as the 1^st^ exon for the *Peg3* locus. On the other hand, U0 exon has a clear exon-intron border in either end, suggesting that this exon is likely connected downstream to the transcripts that start from its upstream regions starting from U1, U2, and U3. None of the transcripts starting from its upstream regions, U1, U2 and U3, are connected to E1. Instead, they all skip the E1 exon, and are directly connected to the E2 exon. This agrees with the fact that the 5′-end sequence of E1 contains a promoter and transcription start site, but not a splicing acceptor site. The exon structures of these newly identified transcripts are summarized in Fig. 2B.

**Figure 3:**
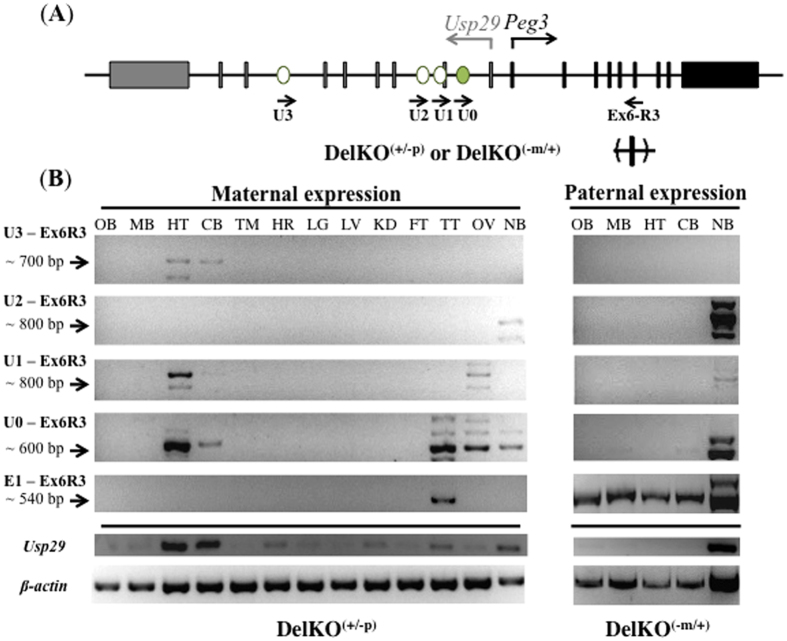
Allele and tissue specificity of the alternative transcripts of *Peg3*. (**A**) A schematic of the mouse *Peg3* locus with RT-PCR primer combinations. Gray and black boxes indicate the exons of *Usp29* and *Peg3*, respectively. The transcriptional direction for each gene is represented with arrows and corresponding colors above the map. The arrows below the map indicate the directionality of primers: Peg3-RT-Ex6-R3 was coupled with U0, U1, U2, and U3 specific primers to amplify the respective alternative exons. The DelKO^(+/−p)^ mutant has a deletion on exon 6 of the *Peg3* paternal allele, enabling the detection of its maternal allele expression. Similarly, the DelKO^(−m/+)^ mutant has a deletion on exon 6 of the *Peg3* maternal allele, enabling the detection of *Peg3* paternal allele expression. (**B**) Allele, tissue, and stage specificity of upstream alternative promoters of *Peg3*. The left RT-PCR panel shows the maternal expression patterns of the identified alternative promoters using total RNA isolated from tissues of DelKO^(+/−p)^ mice: OB (olfactory bulb), MB (midbrain), HT (hypothalamus), CB (cerebellum), TM (thymus), HR (heart), LG (lung), LV (liver), KD (kidney), FT (fat), TT (testis), OV (ovary), and NB (neonate head). The right RT-PCR panel shows the paternal expression patterns using total RNA isolated from tissues of DelKO^(−m/+)^ mice: OB (olfactory bulb), MB (midbrain), HT (hypothalamus), CB (cerebellum), and NB (neonate head). The U3-Ex6R3, U2-Ex6R3, and U1-Ex6R3 primer combinations amplified the upstream alternative 1^st^ exons of *Peg3*, whereas U0-Ex6R3 primer combination amplified a shared upstream exon of *Peg3* to show the expression profile preferred by each upstream exon. The combination of exon 1 and exon 2 primers was used to explore the expression pattern of *Usp29*. Equal amounts of total RNA were used for RT-PCR, which were further normalized and visualized by β-actin expression levels. We repeated this analysis using three independently derived replicates.

Third, the newly identified transcripts U1, U2, and U3 all appear to contain a proper combination of the previously identified downstream exons of *Peg3*, including E2 through E6, based on a subset of the long sequence reads that had been derived from the libraries. Thus, we performed an independent series of RT-PCR experiments to determine the extent of the transcripts starting from U1, U2 and U3, particularly focusing on the 3′-ends of these transcripts. These analyses confirmed that all the transcripts starting from U1, U2 and U3 indeed contain properly spliced downstream exons, including E2 through E9 (Fig. S1). Since the known ORF (Open Reading Frame) of *Peg3* spans from E3 through E9, this further suggests that these transcripts may serve as mRNA templates that produce the PEG3 protein. Fourth, each of the identified transcripts was represented differently compared to the other transcripts in the four libraries of 5′ RACE products based on the number of its sequence reads (Table 1). The U2 represented the highest amount of transcripts (77%, 4267/5473), followed by U1 (8.17%, 447/5473) and U3 (0.09%, 5/5473), according to the results from the third library that had been derived from the WT neonatal brain. The remaining 14.7% (754/5473) products were non-specific sequence reads. This trend was also true for two other libraries derived from neonates, with U2 transcript showing the highest percentage among the newly identified transcripts. Interestingly, this was not the case in the library derived from the hypothalamus of the adult DelKO mice (Table 1 and Fig. S2). Instead, U2 transcript was not detected at all while the remaining U1 and U3 transcripts were well represented. It is relevant to note that the tissue source of this library was the adult hypothalamus, rather than the neonate brain, which was used for the other three libraries. Thus, U2 transcript might be neonatal-specific, which was further tested in the following section (Fig. 3). On a different note, the relative representation of U2 transcript to the known main transcript (E1-E9) was 0.33 to 93%, indicating that the three identified transcripts are overall very minor compared to the primary transcript starting from E1, as shown by the results from the second library. Since these alternative transcripts were also detected from the wild-type animals, they likely represent the genuine transcripts of *Peg3* rather than potential artifacts that could be generated as an outcome of knockout mutations. Scheme I and scheme II (Table 1) are different in terms of the gene-specific primers used for the respective nested PCR scheme as shown by Fig. 2. Even in the case of Scheme II, two different nested primers were also used between two different libraries: a shared upstream exon (U0) as an anchor for the DelKO and WT tissues (Table 1) versus the exon 4 as an anchor for the hypothalamus of adult DelKO (Fig. S2B). Thus, comparison of the results from these two schemes needs a caution. Taken together, this series of 5′ RACE and NGS sequencing confirmed that the *Peg3* locus harbors three alternative 1^st^ exons and promoters that have never been previously described. The three alternative 1^st^ exons and promoters are distributed throughout a relatively large genomic interval (the middle 200-kb region of the *Peg3* imprinted domain), which as of yet has not been well characterized.

### Allele and tissue specificity of the alternative transcripts of *Peg3*

The three alternative transcripts of *Peg3* were further characterized in terms of their allele and tissue specificity using RT-PCR experiments (Fig. 3). For allele specificity, an RT-PCR scheme targeting exon 6 was employed to differentiate the two alleles using the DelKO mutant, which contains a deletion in its exon 6 of *Peg3* (Fig. 3A). The tissues isolated from DelKO^(+/−p)^ and DelKO^(−m/+)^ were used to test the maternal and paternal expression of the alternative transcripts, respectively (Fig. 3B). For tissue specificity, total RNA was isolated from twelve individual tissues of adult mice, including the olfactory bulb (OB), midbrain (MB), hypothalamus (HT), cerebellum (CB), thymus (TM), heart (HR), lung (LG), liver (LV), kidney (KD), fat (FT), testis (TT), and ovary (OV). For stage specificity, total RNA was also isolated from one-day-old neonatal brains (NB), and the results from these neonatal brains were compared with those from the four different parts of the adult brains (OB, MB, HT, CB). Two sets of total RNA from DelKO^(+/−p)^ and DelKO^(−m/+)^ were first reverse-transcribed, normalized with the expression level of an internal control (β-actin), and finally used for the expression analyses of the alternative transcripts.

The results from this series of expression analyses are summarized as follows. First, U3 transcript was detected at very low levels only in the hypothalamus (HT) and cerebellum (CB) of the adult mice, but not in the neonatal brains (NB), indicating its specific expression in HT and CB in the adult stage. This expression pattern was also independently confirmed through the samples from wild-type mice, showing exclusive expression of U3 transcript in the mid-brain and cerebellum (Fig. S3B). More importantly, U3 transcript was detected only in the tissues of DelKO^(+/−p)^, indicating maternal allele-specific expression. Second, U2 transcript appeared to be neonatal-specific, as expression was detected only in the two neonatal brain samples, DelKO^(+/−p)^ and DelKO^(−m/+)^. This also indicated bi-allelic expression of U2 transcript although the expression levels from the paternal allele were much higher than those from the maternal allele. Thus, the expression of U2 transcript is bi-allelic but with a bias toward the paternal allele. Independent analyses also indicated that U2 transcript is not detectable in 14.5-dpc embryos and placentas, further confirming its neonatal-specific expression (Fig. S3B). Third, the expression of U1 transcript was detected in the hypothalamus (HT) and also in the ovary (OV). Interestingly, U1 transcript was present in the hypothalamus (HT) of DelKO^(+/−p)^, and absent from that of DelKO^(−m/+)^, thus indicating a maternal allele-specific expression pattern for U1, similar to U3 transcript. The expression of U1 transcript in ovary (OV) was further investigated by performing an independent RT-PCR and also 5′ RACE experiments using mature oocytes. U1 transcript was not detected at all in mature oocytes (data not shown), indicating that the observed expression of U1 transcript likely originated from either the early-stage oocytes or the somatic cells of ovary. Fourth, a series of RT-PCR surveying U0 exon detected the expression from the hypothalamus (HT), cerebellum (CB), and ovary (OV) of DelKO^(+/−p)^ and also from the neonatal brains (NB) of both DelKO^(+/−p)^ and DelKO^(−m/+)^. This is reasonable since the three alternative transcripts, U1, U2, and U3, which contain the U0 exon, were detected from the same tissues that show the U0 transcript. Interestingly, the same RT-PCR also detected the expression from the testis (TT), which was not detected by the previous RT-PCR surveying U1, U2, U3 transcripts. This suggests that additional unknown transcripts may exist in testis. Multiple minor PCR products observed throughout this expression analysis (U3, U2, U1 and U0) were confirmed to result from splicing variations (Fig. 3B). Taken together, this series of expression analyses concluded that U1 and U3 transcripts are maternal allele-specific whereas U2 is bi-allelic with a bias toward the paternal allele. In addition, the main expression sites of these alternative transcripts include the hypothalamus for U1 and U3 and the neonatal brain for U2.

### DNA methylation analysis of the alternative promoters of *Peg3*

**Figure 4:**
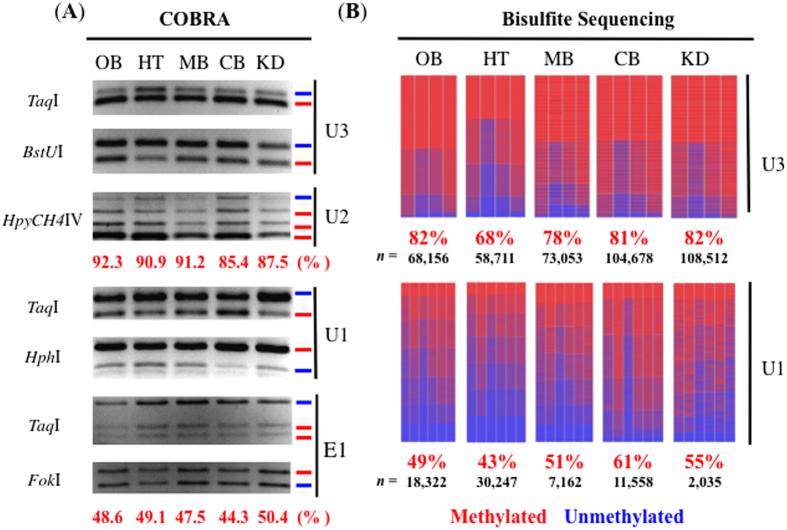
DNA methylation analysis of alternative promoters of *Peg3*. (**A**) DNA methylation analysis of the E1, U1, U2, and U3 promoter regions. Genomic DNA was purified from the different parts of the brain (olfactory bulb, hypothalamus, midbrain, cerebellum) and kidney of a two-month-old female mouse, and then used for bisulfite conversion. The bisulfite-converted DNAs were subsequently amplified with PCR using specific primer sets designed for each promoter region (Table S1). The amplified PCR products were analyzed by COBRA. The restriction enzymes used for each digestion is shown on the left side of the panel, while the right side of the panel indicates the promoter regions under investigation. The red lines indicate methylated DNA whereas the blue lines indicate unmethylated DNA. The methylation levels of U2 and E1 promoter regions were calculated using the ImageJ software for three independent trials. (**B**) Quantitative methylation analysis of U1 and U3 promoter regions. The bisulfite-converted DNAs were amplified with PCR and used for NGS-based deep sequencing to obtain the methylation level for each adult mouse tissue. A single red box and a blue box indicate a single read for methylated and an unmethylated CpG site, respectively. Each column in the methylation array represents a single CpG site for the respective tissue sample. The U3 and U1 promoter regions represent DNA methylation changes observed from four and six CpG sites, respectively. The overall percentage of methylation is indicated at the bottom of each tissue for comparison. The number of reads are represented by the (*n*) underneath each tissue sample.

DNA methylation status of the three alternative promoters was further analyzed given their allele and tissue-specific expression patterns. Genomic DNA was first isolated from four different parts of the brain: olfactory bulb (OB), hypothalamus (HT), midbrain (MB), cerebellum (CB), as well as kidney (KD) of a wild-type female adult mouse (Fig. 4). The isolated DNA was treated with the bisulfie conversion protocol^26^, and the converted DNA was used as a template for PCR amplification. Four different regions were targeted for DNA methylation analyses: E1, U1, U2 and U3 promoter regions. The amplified PCR products were analyzed first by COBRA (Combined Bisulfite Restriction Analysis^27^), and later by individual sequencing (Table 2 and Fig. 4).

The promoter region of an imprinted gene usually derives 50% methylation level due to its allele-specific methylation status: one allele is methylated while the other allele is unmethylated. The allele-specific methylation pattern is very uniform and complete among individual CpG sites in a given promoter and also among individual tissues. This is the case for E1 promoter, which is part of the 4-kb Peg3-DMR, showing 50% methylation level among all the tissues tested. On the other hand, the methylation levels and patterns of the three alternative promoters are quite different from those from E1. First, U1 promoter also showed around 50% methylation levels, but the levels were variable among the tissues, ranging from 43% in the hypothalamus (HT) to 61% in the cerebellum (CB). Detailed inspection further indicated that the methylation level and pattern of each CpG site were also variable compared to that of the remaining CpG sites. This is quite different from the uniform and complete pattern observed from E1 promoter, thus suggesting that the DNA methylation on U1 promoter may not be of allelic origin. The same may be the case for U3 promoter, as its individual CpG sites displayed fluctuating levels of DNA methylation. Compared to the U1 promoter, the overall methylation levels of U3 promoter were greater, ranging from 68% in the hypothalamus (HT) to 82% in the olfactory bulb (OB) and kidney (KD). This is somewhat consistent with the higher expression levels observed from U1 transcript than those from U3 transcript (Fig. 3B). Also, the methylation levels of both U1 and U3 promoters in the hypothalamus were the lowest among the tissues tested, which also agrees with the fact that the expression of U1 and U3 transcripts were observed only from the hypothalamus. Finally, U2 promoter displayed the greatest DNA methylation levels among the alternative promoters, ranging from 85.4% in the cerebellum (CB) to 92.3% in the olfactory bulb (OB). This is again consistent with the fact that the expression of U2 transcript was not detectable at all in the adult tissues (Fig. 3B). Overall, this series of analyses concluded that the methylation levels and patterns of the alternative promoters are variable among individual tissues and also among individual CpG sites. Thus, the DNA methylation on the alternative promoters is somewhat different from the typical pattern of allelic origin, although the expression of U1, U2, and U3 transcripts appears to be biased in either the maternal or the paternal allele. Nevertheless, this aspect of the results is inconclusive at the moment, which requires further investigation in the near future.

### Identification of the alternative 1^st^ exon and promoter of human *PEG3*

**Figure 5:**
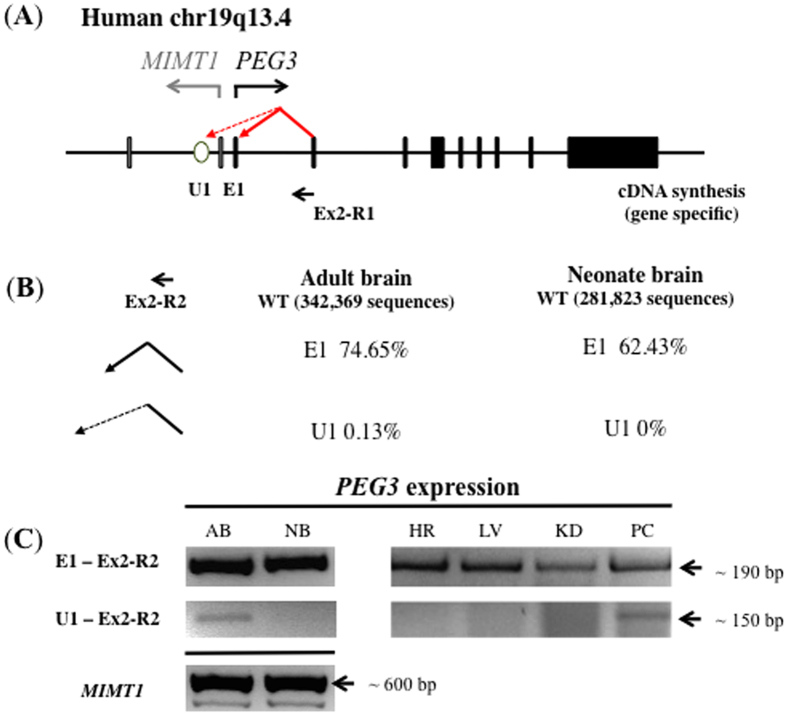
Upstream alternative promoter of human *PEG3*. (**A**) A schematic of the human *PEG3* locus with RT-PCR primer combinations. Gray and black boxes indicate the exons of *MIMT1* and *PEG3*, respectively. Transcriptional direction for each gene is represented using arrows with corresponding colors. The black arrow indicates the directionality of Ex2-R1, the primer used for cDNA synthesis. The open oval represents the alternative 1^st^ exon U1. (**B**) 5′ RACE analysis for upstream exons of *PEG3*. The Ex2-R2 indicates the anchoring primer used for nested PCR after 5′ RACE. This primer was coupled with U1 and E1 specific primers to amplify the respective exons. The percentage of transcripts preferred by the adult human brain was calculated by counting the sequences specific for U1 and E1. (**C**) RT-PCR analysis of upstream alternative exons in human tissues. The RT-PCR panel shows the expression patterns of *PEG3* using cDNA from AB (adult brain), NB (neonate brain), HT (adult heart), LV (adult liver), KD (adult kidney) and PC (adult placenta). U1-Ex2R2 and E1-Ex2R2 primer combinations amplified the upstream exons of *PEG3* to show the expression profile preferred by each 1^st^ exon. The Ex1-Ex2 primer combination amplified the expression pattern of *MIMT1* in the AB and NB. Equal amounts of total RNA were used for RT-PCR, which were normalized by β-actin expression levels.

The genomic structure of the *Peg3* imprinted domain is well conserved among individual mammals, including humans^2^,^8^. Thus, we tested the presence of potential alternative 1^st^ exons and promoters of human *PEG3*. This test also used a similar strategy involving 5′ RACE and NGS sequencing as described above (Fig. 5A). The total RNA isolated from adult and neonate brain tissues was first reverse-transcribed with a gene-specific primer targeting exon 2 of human *PEG3* (Ex2-R1), and later these cDNA fragments were further amplified with a set of nested primers (Ex2-R2 and Ex2-R3). The two amplified libraries were sequenced using a NGS platform. The results are summarized in Fig. 5B. According to the results, about 74% of the sequence reads from the adult brain (255, 571/342, 369) belong to the known 1^st^ exon of human *PEG3* (E1). On the other hand, 0.13% of the sequence reads (433/342, 369) derived from a 120-bp region that is located 2.4-kb upstream region of E1 exon. Yet, these sequences were not connected to the E1 exon, but directly connected to the 2^nd^ exon of *PEG3* (E2). Also, this set of cDNA sequences shows a similar pattern as seen from the three alternative promoters of mouse *Peg3*, as a clear exon-intron border exists in their 3′-end sequences but not in their 5′-end sequences. Therefore, this exon is predicted to be an alternative 1^st^ exon of human *PEG3*, thus named U1. Interestingly, this U1 exon was not detected in the library derived from the neonate brain, thus indicating an adult-specific expression (Fig. 5B). To further confirm this possibility, we also performed a set of RT-PCR testing the expression pattern of U1 exon (Fig. 5C). As expected, the expression levels of the human *PEG3* transcript starting from E1 exon were very high in both adult and neonatal brains (AB and NB), and also in the other adult tissues, including heart (HR), liver (LV), kidney (KD) and placenta (PC). On the other hand, the expression of the human *PEG3* transcript starting from U1 was detected at very low levels only from the adult brain (AB) and placenta (PC). This low level of expression in adult brain agrees with the initial detection of this exon from the library that had been derived from adult brain. Also, this appears to be somewhat similar to the mouse U1 and U3 transcripts since they are also expressed only in the adult stage. Overall, this series of 5′ RACE and expression analyses confirmed that human *PEG3* also harbors an alternative 1^st^ exon and promoter. Thus, alternative 1^st^ exons and promoters may be evolutionarily conserved features that are associated with the mammalian *Peg3* domain.

## Discussion

In the current study, we have identified three alternative promoters for mouse *Peg3* and one alternative promoter for human *PEG3*. These alternative promoters are localized within the 200-kb upstream regions of mouse and human *PEG3*, which are well conserved among mammals in terms of genomic structure. In mouse, two of these promoters, U1 and U3, derive maternal-specific expression whereas the remaining promoter, U2, derives bi-allelic expression of *Peg3* with a paternal allele bias. These promoters are also tissue and stage-specific: U1 and U3 transcripts are detected in the hypothalamus of adult mice whereas U2 transcript only in neonatal-stage brains. In humans, U1 transcript is detected at relatively very low levels in adult brain and placenta. Overall, the identification of alternative promoters in both mouse and human suggest that these alternative promoters may be functionally selected features for the *Peg3* imprinted domain during mammalian evolution.

**Figure 6:**
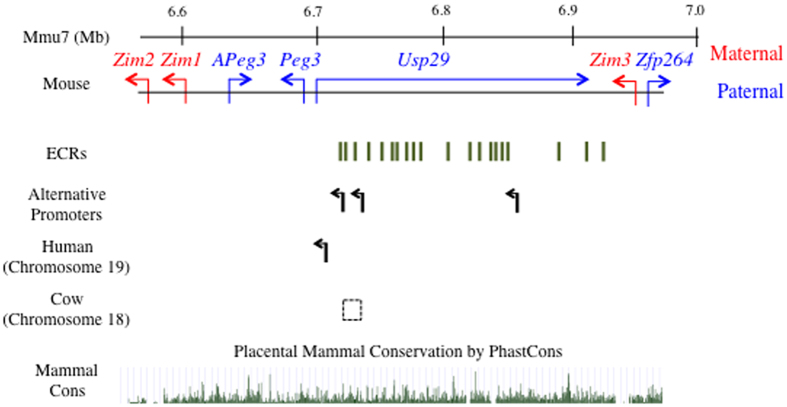
Evolutionary conservation of mammalian *Peg3* domains. The 500-kb genomic interval of the *Peg3* imprinted domain is represented using the mouse region as a representative locus. The blue and red colors indicate paternally expressed (*APeg3, Peg3, Usp29, Zfp264*) and maternally expressed genes (*Zim1, Zim2, Zim3*), respectively. Transcriptional direction for each gene is represented using arrows with corresponding colors. The evolutionarily conserved regions (ECRs) are shown using green lines. The upstream alternative promoters for *Peg3* in mouse chromosome 7 are shown using three black arrows. The upstream alternative promoter for *PEG3* in human chromosome 19 is shown using a black arrow. The dotted box indicates the genomic region responsible for stillbirth in cows (64, 325, 122-64, 431, 506). The evolutionary conservation of the mammalian *Peg3* domains is indicated by the mammal conservation plot, which is represented in green.

The current study has identified alternative promoters for human and mouse *PEG3* with a strategy involving 5′ RACE and NGS-based deep sequencing experiments (Figs 2 and 5). The identified alternative promoters are all located within the 200-kb genomic interval that is upstream of the *Peg3* locus (Fig. 6). This 200-kb genomic interval has been well conserved during mammalian evolution although this region lacks an obvious ORF^8^. According to recent studies, this genomic interval contains 18 evolutionarily conserved regions (ECRs), and these ECRs are all associated with H3K4me1 and/or H3K27ac^9^. Thus, these ECRs are thought to be potential enhancers. Some of the alternative promoters are in fact closely associated with these ECRs. For instance, U2 is localized between ECR3 and ECR4, whereas U3 is located nearby ECR18. This may be an indication that some of the ECRs are enhancers for these alternative promoters. On the other hand, a similar series of inspection was unable to confirm the association of any histone marks, such as H3K4me3, with these alternative promoters, which might be caused by their very low abundance. Recent studies from the bovine genome also revealed that a deletion of a 20-kb genomic interval encompassing U2 promoter might be responsible for the stillbirths observed among the calves that had been derived through artificial insemination^28^. This observation is intriguing in that U2 promoter is very specific in neonatal-stage brains (Fig. 3). This further suggests that these alternative promoters may play very unique, but critical roles for the transcription control of *Peg3*. Overall, the identification of alternative promoters in mouse and human *PEG3* appears to provide an answer for a long-standing, puzzling question: why is the middle 200-kb genomic region of the *Peg3* domain preserved so well during mammalian evolution? This region most likely harbors several *cis*-regulatory elements critical for the *Peg3* domain, and one set of these should be the newly identified alternative promoters for human and mouse *PEG3*.

The three alternative promoters of mouse *Peg3* are quite interesting in terms of their expression patterns (Fig. 3). First, one of the main expression sites shared among the three promoters is the hypothalamus. It is well known that *Peg3* is highly expressed in the hypothalamus^1^,^3^,^10^,^22^, and the *in vivo* functions of *Peg*3 are closely associated with several roles played by this part of brain, such as milk provision and maternal-caring behavior^10^. The similar expression pattern shared between the new alternative promoters (U1, U3) and the known major promoter (E1) suggests that U1 and U3-driven transcripts likely play similar roles as the main transcript by E1 promoter. This further suggests that unknown *cis*-regulatory elements may exist for this hypothalamus-specific expression of these promoters. Second, U2 promoter shows a highly sensitive, stage-specific expression, showing its expression exclusively in one-day-old neonates, but not in 14.5-dpc embryos or adult tissues (Fig. S3). Thus, this promoter might be functional only during a particular stage when neonates’ brain is known to undergo a series of major organization processes, especially in the hypothalamus region^29^,^30^. Therefore, the main roles of this promoter might be associated with many changes occurring during this transition period of brain development. Third, two of the alternative promoters, U1 and U3, show maternal allele-specific expression patterns. It is puzzling at the moment why these alternative promoters derive maternal-specific expression for the paternally expressed *Peg3* in the hypothalamus. One possible explanation would be that some of cell populations within the hypothalamus might require additional dosage of *Peg3*. As a consequence, *Peg3* might be bi-allelic in those cell populations: maternal by U1 and U3 and paternal by E1 promoter. Since the alternative promoters reside within the paternally expressed *Usp29* transcriptional region, transcriptional interference by *Usp29* could be another possible explanation for the allele-specific expression patterns observed by U1, U2 and U3^31^. In spite of the observed allele-specific expression, however, DNA methylation pattern suggests that it is unlikely of allelic origin (Fig. 4). Nevertheless, the absence of sequence polymorphisms in the alternative promoter regions between different subspecies of the mouse hampered the inquiry of allele-specific DNA methylation. Overall, the newly identified alternative promoters exhibit very interesting expression profiles, which have not been observed before. Thus, characterizing these promoters should provide additional insights regarding the *in vivo* function of *Peg3*.

Alternative promoters have also been identified from other imprinted domains, such as *Grb10*, *Snrpn*/*Ube3a*, *Gnas* and *Zac1* domains. Interestingly, the two alternative promoters for a given locus tend to be opposite in terms of their allele-specific expression. The examples include maternal and paternal-specific promoters in *Grb10* and *Gnas*^15^,^16^,^17^,^20^. Because of the current study, this list includes the maternal and paternal-specific promoters for the *Peg3* locus as well. According to the observations from other domains, several *in vivo* roles are possible for the alternative promoters of the *Peg3* locus. First, the two promoters for *Grb10* have two complementary expression patterns. The paternal and maternal promoters of *Grb10* are responsible for its expression in neuronal and non-neuronal cells, respectively^32^. Thus, this might be feasible for the alternative promoters for *Peg3*: the paternal and maternal promoters could function in different population of cells within the hypothalamus. Second, one upstream alternative promoter for the *Snrpn* locus is also involved in regulating the allele-specific expression of the nearby gene, *Ube3a*, which is located 500-kb downstream of *Snrpn*. This particular promoter derives the paternal-specific transcription of a long non-coding *Ube3a-ATS*^12^. Furthermore, this antisense transcript is thought to be responsible for maintaining the maternal-specific expression of *Ube3a*^13^,^14^. The alternative promoters of *Peg3* might also play similar roles. In particular, U3 promoter is located 160-kb upstream of *Peg3*, yet the transcription initiated from this promoter extends all the way downstream to the *Peg3* locus. It is possible that the transcription starting from U3, particularly from the maternal allele, might be related to the expression of two maternally expressed downstream genes of *Peg3*, *Zim1* and *Zim2*. Besides these two possible roles, the alternative promoters might be also involved in the DNA methylation setting for the Peg3-DMR during oogenesis. A similar situation is known to occur in the *Gnas*, *Snrpn*, and *Zac1* loci^13^,^14^,^17^,^19^. According to a deep transcriptome sequencing study of the mouse oocytes, several alternative promoters were identified for individual imprinted genes including *Peg3*^19^. However, none of the three alternative promoters of the *Peg3* locus seem to be functional and thus detectable in mature oocytes, based on our preliminary results obtained so far (data not shown). Thus, these promoters might not play a similar role in DNA methylation setting as seen in these loci. Overall, the information from the other imprinted domains appears to provide several hints regarding the potential roles that might be played by the alternative promoters of *Peg3*. Thus, it should be very interesting to pursue these aspects of the identified alternative promoters in the near future.

## Materials and Methods

### Ethics Statement

All the mouse experiments were performed in accordance with National Institutes of Health guidelines for care and use of animals and also approved by the Louisiana State University Institutional Animal Care and Use Committee (IACUC), protocol #13-061.

### Generating the mutant strains for *Peg3*

The current study used the KO2 and DelKO mutant mouse strains derived from KO2-Neo and CoKO strains, respectively. The strain for the CoKO allele of *Peg3* was made using a targeted ES cell from the EUCOMM (European Conditional Mouse Mutagenesis program), and this strain has been maintained in the lab^23^. The strain for the KO2-Neo allele of *Peg3* was made using the targeted ES cells from Darwin Transgenic Mouse Core Facility of Baylor College of Medicine, and this strain has been maintained in the lab (H. He *et al*., unpublished). The CoKO mouse strain was crossed with the Rosa26-FLP line from Jackson Lab (Stock No. 009086, B6.129S4-*Gt* (ROSA)*26Sor*^*tm1(FLP1)Dym*^/RainJ) to allow for FLP-mediated recombination, generating the FlipKO allele. Both the FlipKO and KO2-Neo strains were crossed with the Zp3-Cre line from the Jackson Lab (Stock No. 003651, C57BL/6-Tg (Zp3-cre) 93Knw/J). The mutagenesis through these breeding derived the DelKO and KO2 strains for the *Peg3* locus^22^,^24^. The following primer sets were used for genotyping of these strains: the deletion and detection of the expression cassette for FlipKO, Peg3-CoKO-F (5′-ATGAGTCTCGATCCAGGTATGCC-3′) and LoxR (5′-TGAACTGATGGCGAGCTCAGACC-3′), Peg3-5arm (5′-CCCTCAGCAGAGCTGTTTCCTGCC-3′) and Peg3-Lar3 (5′-CAACGGGTTCTTCTGTTAGTCC); the deletion and detection of the expression cassette for DelKO, Peg3-5arm (5′-CCCTCAGCAGAGCTGTTTCCTGCC-3′) and LoxR (5′-TGAACTGATGGCGAGCTCAGACC-3′), Peg3-5arm (5′-CCCTCAGCAGAGCTGTTTCCTGCC-3′) and Peg3-rev (5′-ACCCCATTCTCATCAGCTCCAGAG-3′), the deletion of exon 1 for *Peg3* and *Usp29*, bac2082-F (5′-ACAACCCGGAGTTTTAGCAGAC-3′) and bac6710-R (5′-GGATGTAAGATGGAGGCACTGT-3′); the presence of Zp3-Cre, Zp3-cre-F (5′-TAGGAATCACGTGGAGTGTCT-3′) and oIMR1085 (5′-GTGAAACAGCATTGCTGTCACTT-3′). DNA was isolated from ear or tail snips after incubating the tissues at 65 °C with the tail lysis buffer (50 mM Tris-Cl at pH 8.0, 100 mM EDTA at pH 8.0, 250 mM NaCl, 1% SDS, 20 μg/mL Proteinase K). PCR premix kit (Intron Biotech) was used for genotyping at the following conditions (step 1, 95 °C–2 min; step 2, 95 °C-30 sec, 60 °C-30 sec, 72 °C-60 sec for 33 cycles; step 3, 72 °C-7 min). The information regarding individual primer sequences is also available (Table S1). All animal studies were approved by the Louisiana State University Institutional Animal Care and Use Committee (IACUC) and were performed in accordance with approved guidelines and regulations of the LSU Division of Laboratory Animal Medicine, Baton Rouge, Louisiana, USA.

### 5′ RACE and sequencing

Total RNAs were isolated from adult mouse hypothalamus and one-day-old mouse neonate heads using the Trizol RNA isolation kit (Invitrogen). Total RNAs for normal adult and neonate human brains were purchased from BioChain (cat: R1234035-50; R1244035-50). The total RNA (2.5–5 μg) was mixed with gene-specific primers substituting random primers: Ex2-R1 (5′-AGTCTTCCTCTTGCCAGTTGTC-3′) for KO2 mice, Ex6-R2 (5′-CCAAAATGTGGTCTTGACATCACAG-3′) for DelKO mice, and Ex2-R1 (5′-TCCCTCTTCCTCTCGCCAGTCG-3′) for human, and reverse-transcribed using the M-MuLV reverse transcriptase (New England Biolabs, cat: M0253S). The cDNA products were purified using phenol chloroform extraction and ethanol precipitation. The 5′-ends of the purified cDNA were further modified by the tailing reaction using dGTPs and terminal deoxynucleotidyl transferase according to the manufacturer’s protocol (New England Biolabs, cat: M0315S). The tailed cDNA was amplified using two primers: the tail long primer (5′-GGTTGTGAGCTCTTCTAGATCCCCCCCCCCCCNN-3′) and internal gene-specific primers: Ex2-R2 (5′-TCCTCTTGCCAGTTGTCTCCAA-3′) for KO2 mice, Ex6-R3 (5′-ATGTGGTCTTGACATCACAGGAAGA-3′) for DelKO mice, and Ex2-R2 (5′-CTTCCTCTCGCCAGTCGTCTCC-3′) for human. The amplified cDNA was re-amplified with a set of nested primers: the tail out primer (5′-GGTTGTGAGCTCTTCTAGA-3′) and additional internal gene-specific primers as anchors. The PCR products were further purified and sequenced using next generation sequencing (NGS) for analysis of 5′ RACE products^25^.

### RNA isolation and RT-PCR analysis

Total RNA was isolated from the olfactory bulb, midbrain, hypothalamus, cerebellum, thymus, heart, lung, liver, kidney, fat, testis, 14.5-dpc placenta and ovary from adult male and female mice, heads of one-day-old neonates, and 14.5-dpc embryos using a commercial kit (Trizol reagent, Life technologies, cat: 15596018) according to the manufacturer’s protocol. Total RNAs for normal human adult brain and the neonate brain were purchased from BioChain (cat: R1234035-50; R1244035-50). The total RNA was then reverse-transcribed using the M-MuLV reverse transcriptase (NEB, cat: M0253S). A set of normalized cDNA for human heart, liver, kidney, and placenta was obtained from BioChain as well. The cDNA fragments were used as a template for PCR amplification (Maxime PCR Premix Kit, Intron Biotech) to check for mRNA transcripts at the following conditions (step 1, 95 °C-2 min; step 2, 95 °C-30 sec, 60 °C-30 sec, 72 °C-60 sec for 36 cycles; step 3, 72 °C-7 min). The information regarding individual primer sequences is also available (Table S1).

### DNA methylation analysis

DNA was first isolated from the hypothalamus, olfactory bulb, midbrain, cerebellum, and kidney of WT adult mice, and subsequently treated with the bisulfite conversion protocol using the EZ DNA methylation kit (Zymo Research, cat: D5002). The converted DNA was used for PCR amplification of the upstream exons regions E1, U1, U2, and U3 (Maxime PCR Premix Kit, Intron Biotech). The following primer combination was used to amplify the upstream U1 promoter: Bis-Peg3-RACEF1-F1 (5′-GTTGGGAATGGAAAGTTTAAAGATAAA-3′) and Bis-Peg3-RACEF1-R1 (5′-AAAATCAAAACTACACCAAACATACAAC-3′). Upstream U2 promoter was amplified by using the following primer combination: ECR4-Bis-a (5′-ATTGGTTTATAGTTAGGGAAGGAAGTAGT-3′) and ECR4-Bis-b (5′-AAATCTCTCTAAAACATAATACTATTCTAT-3′). The following primer combination was used to amplify the upstream U3 promoter: Bis-Peg3-RACEF5-F (5′-GTAGGTAGATAATTTATTGGATAAAGAGTT-3′) and Bis-Peg3-RACEF5-R (5′-CTTTCTTTCTCTCTTTCTTTATACATATAT-3′). The upstream E1 promoter was amplified by using the following primer combination: mPeg3-pro-bis-a.1 (5′-GTTTTTGTAGAGGATTTTGATAAGGAG-3′) and mPeg3-pro-bis-b (5′-CACCCCAAACACCATCTAAACTCTACAAAC-3′). Each PCR product was further analyzed by restriction enzyme digestion-based COBRA (COmbined Bisulfite Restriction Assay) and sequencing^26^,^27^. For the COBRA analysis, PCR products were digested with various restriction enzymes (NEB). Each PCR product was also used for next-generation sequencing (NGS). Detailed information regarding oligonucleotide sequences, and COBRA is also available (Table S1).

## Additional Information

**How to cite this article**: Perera, B. P. U. and Kim, J. Alternative promoters of *Peg3* with maternal specificity. *Sci. Rep*. **6**, 24438; doi: 10.1038/srep24438 (2016).

## Supplementary Material

Supplementary Information

## Figures and Tables

**Table 1:** Summary of NGS-based sequencing of 5′ RACE libraries from mouse *Peg3*.

Tissues (genotype)	% of specific reads for different transcripts			
	Neonatal brain (KO2)	Neonatal brain (WT)	Neonatal brain (WT)	Adult hypoth (DelKO)
RACE scheme (# of total read)	I (575)	I (1843)	II (5473)	II (1197)
E1-E2-9	6.60	93.54	–	–
U1-U0-E2-9	0.00	0.00	8.17	78.96
U2-U0-E2-9	67.83	0.33	77.96	0.00
U3-U0-E2-9	0.00	0.00	0.09	7.60

**Table 2:** Summary of the alternative promoter positions for DNA methylation analysis.

Upstream promoter/exon (Chr: 7)	Position (NCBI37/mm9) direction towards *Peg3*	Position used for DNA methylation analysis
U3	5′ (~157 bp) – 6, 846, 590 3′	5′ 6, 846, 749 – 6, 846, 565 3′
U2	5′ (~ 79 bp) – 6, 709, 554 3′	5′ 6, 760, 018 – 6, 759, 695 3′
U1	5′ (~178 bp) – 6, 703, 769 3′	5′ 6, 704, 151 – 6, 703, 900 3′
U0	5′ 6, 699, 550 – 6, 699, 412 3′	N/A
E1	5′ (~164 bp) – 6, 682, 968 3′	5′ 6, 846, 749 – 6, 846, 566 3′
