# Supplementary material for: Alternative promoters of Peg3 with maternal specificity
Source: Sci Rep. 2016 Apr 14;6:24438. doi: 10.1038/srep24438 (PMC4830991; doi:10.1038/srep24438)
Supplement: Supplementary Information [file srep24438-s1.pdf]

Alternative promoters of *Peg3* with maternal specificity.

Bambarendage P.U. Perera, Joomyeong Kim*.

Department of Biological Sciences, Louisiana State University,
Baton Rouge, LA 70803, USA.

Correspondence should be forwarded to:
jkim@lsu.edu, 225-578-7692(ph), or 225-578-2597(fax)

Fig.S1_Perera

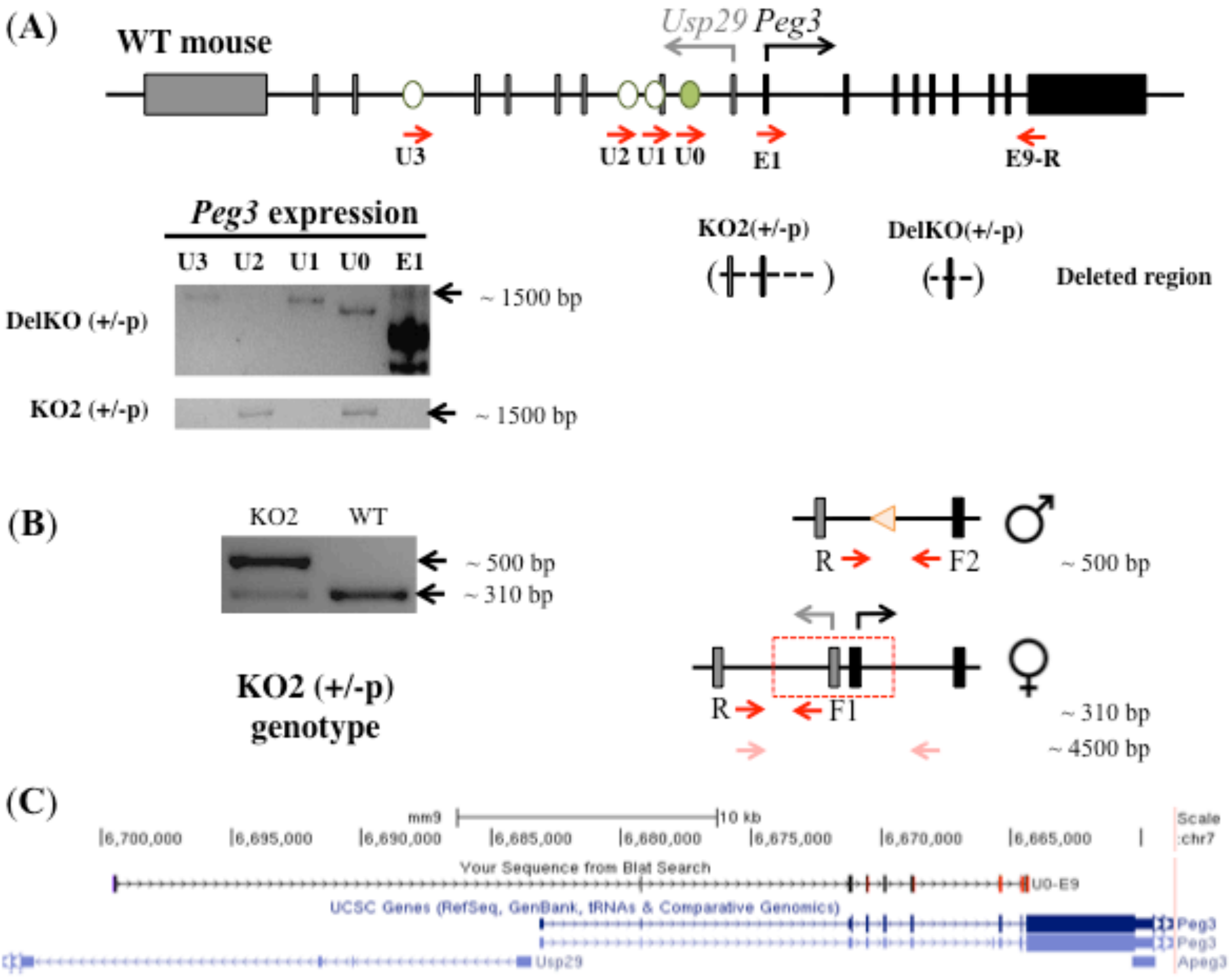

Figure Legends

Figure S1. RT-PCR analysis of alternative exons in the mouse brain. (**A**) A schematic of the mouse *Peg3* locus with RT-PCR primer combinations. Gray and black boxes indicate the exons of *Usp29* and *Peg3*, respectively. The red arrows indicate the directionality of primers: E9-R coupled with E1, U0, U1, U2, and U3 specific primers to amplify the respective exons. The deleted *Peg3* exons corresponding to KO2(+/-p) and DelKO(+/-p) mutant alleles are shown using parentheses and dashed lines. The RT-PCR panel indicates the expression patterns of *Peg3* using total RNA isolated from DelKO(+/-p) adult hypothalamus and KO2(+/- p) neonate brain tissues. The U3, U2, and U1 represent primer combinations targeting the upstream 1st exons of *Peg3* with E9-R primer combination; U0 targets a shared upstream exon of *Peg3* with the E9-R primer, while E1 targets the main promoter of *Peg3* with the E9-R primer, to show the expression profile preferred by each exon. (**B**) Genotyping of the paternally transmitted KO2 allele. The schematic represents the mouse locus for the paternal and maternal alleles of *Peg3*. The arrows indicate the primer combinations used for PCR amplification. The dotted box represents the deleted region corresponding to the bidirectional promoter. (**C**) Structural map of the upstream alternative exons of *Peg3*. The blue rectangles represent *Peg3*, *Usp29*, and *APeg3* exon structures within the 6658776-6703943 genomic region of mouse chromosomes 7, with arrows indicating the respective transcriptional directions. The U0-E9 exon structure represents the genomic region transcribed by the shared upstream alternative exon of *Peg3*, with arrows indicating its transcriptional direction. The UCSC genome browser was used to visualize the exon structure of U0-E9.

Fig.S2_Perera

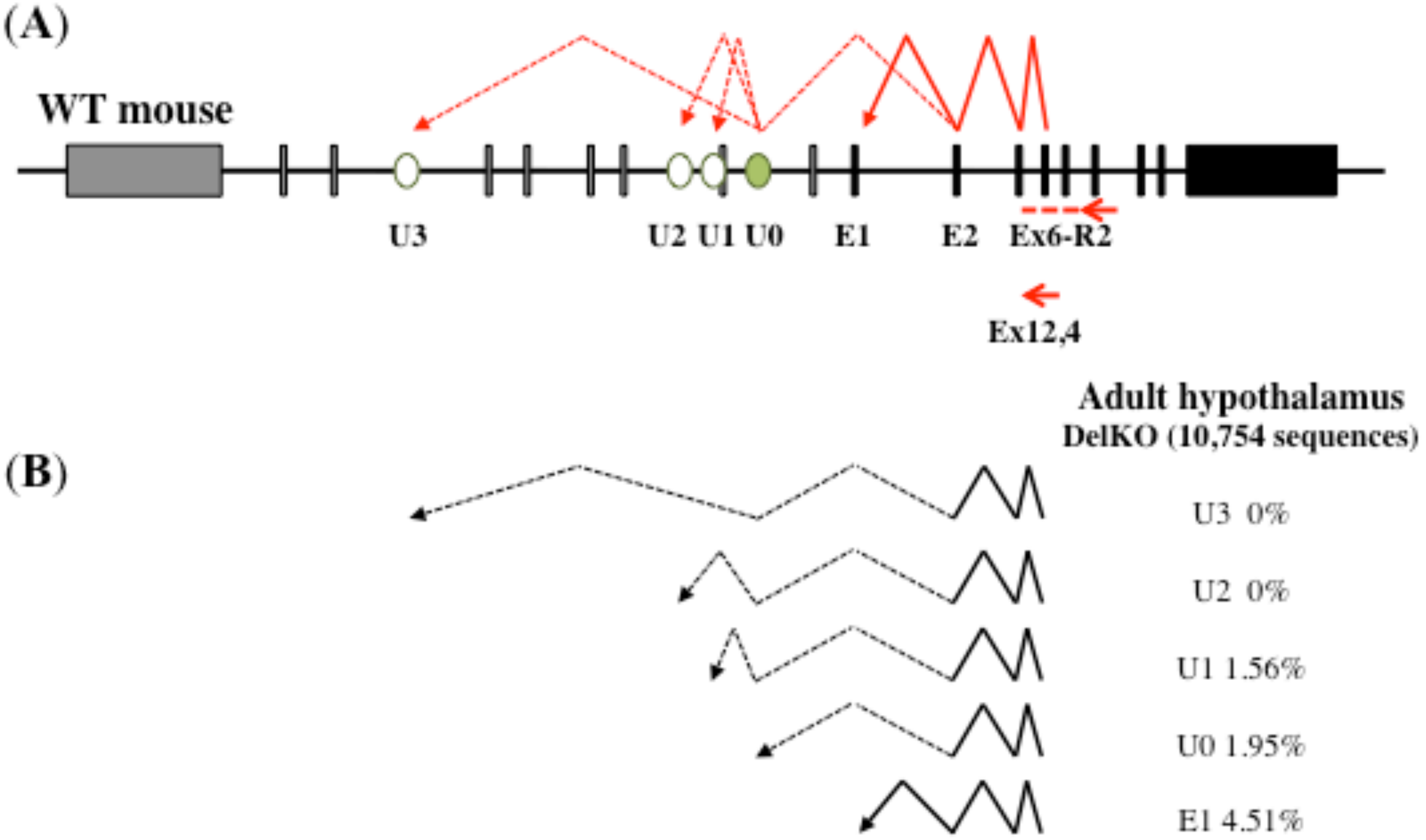

Figure Legends

Figure S2. Alternative transcripts determined by 5' RACE. (**A**) Map of the mouse *Peg3* locus. Gray and black boxes indicate the exons of *Usp29* and *Peg3*, respectively. Transcriptional direction for each gene is represented using arrows with corresponding colors. A solid red arrow indicates the position of the first exon of *Peg3*, labeled E1, followed by dotted arrows to indicate the position of upstream alternative exons. The ovals represent upstream exons U0, U1, U2, and U3, respectively. The extended arrow shows the gene-specific primer Ex6-R2 used for DelKO(+/-p) mouse hypothalamus cDNA synthesis. (**B**) Percentage of *Peg3* alternative transcripts identified from adult mouse hypothalamus. The Ex12,4 indicates the anchoring primer used for nested PCR. The percentage of transcripts preferred by DelKO(+/-p) adult mouse hypothalamus was calculated by counting the sequences specific for E1,U0, U1, U2, and U3.

Fig.S3_Perera

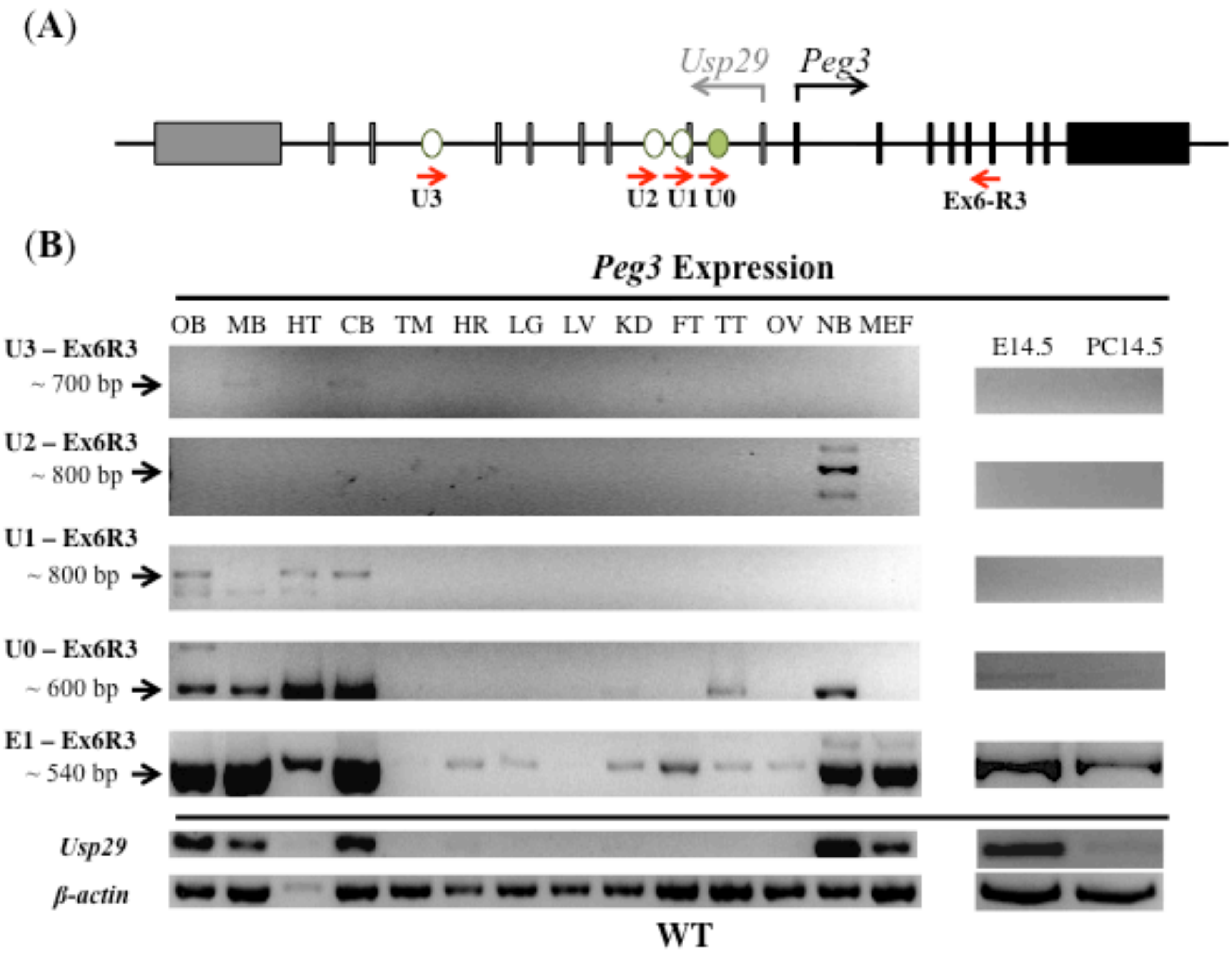

Figure Legends

Figure S3. RT-PCR analysis of alternative exons in wild-type mouse tissues. (**A**) A schematic of the mouse *Peg3* locus with RT-PCR primer combinations. Gray and black boxes indicate the exons of *Usp29* and *Peg3*, respectively. Transcriptional direction for each gene is represented using arrows with corresponding colors. The red arrows indicate the directionality of primers: Ex6-R3 coupled with E1, U0, U1, U2, and U3 specific primers to amplify the respective exons. (**B**) Allele, tissue, and stage-specificity of upstream alternative exons of *Peg3*. The RT-PCR panel shows the expression patterns of *Peg3* using total RNA isolated from tissues of wild-type mice: OB (olfactory bulb), MB (midbrain), HT (hypothalamus), CB (cerebellum), TM (thymus), HR (heart), LG (lung), LV (liver), KD (kidney), FT (fat), TT (testis), OV (ovary), NB (neonate brain), MEF (mouse embryonic fibroblasts), E14.5 (Embryo14.5-dpc.), and PC14.5 (Placenta 14.5-dpc). U3-Ex6R3, U2-Ex6R3, and U1-Ex6R3 primer combinations target the upstream 1st exons of *Peg3*; U0-Ex6R3 primer combination targets a shared upstream exon of *Peg3,* whereas E1-Ex6R3 primer combination targets the main promoter of *Peg3* to show the expression profile preferred by each exon. The combination of exon 1 and exon 2 primers was used for the expression pattern of Usp29. The β-actin expression profile serves as a control to visualize the relative mRNA levels.

Supplementary Table 1. Primer sets used for 5'RACE. RT-PCR, and genotyping experiments.

Locus	Name	Sequence (5' -> 3')	Primer set	Size (bp)	*Position (mm9, NCBI Build 37)
**Primers used for genotyping**					
FlipKO (genotyping)	Peg3-CoKO-F<br>Lox-R	ATGAGTCTCGATCCAGGTATGCC<br>TGAACTGATGGCGAGCTCAGACC	1st primer	≅290	mChr7: 6,669,691-unknown
FlipKO (genotyping)	Peg3-5arm<br>Peg3-rev	CCCTCAGCAGAGCTGTTTCCTGCC<br>ACCCCATTCTCATCAGCTCCAGAG	2nd primer	≅700	mChr7: 6,669,691-6,668,412
DelKO (genotyping)	Peg3-5arm<br>Peg3-LoxR	CCCTCAGCAGAGCTGTTTCCTGCC<br>TGAACTGATGGCGAGCTCAGACC	1st primer	≅600	mChr7: 6,669,691-unknown
DelKO (genotyping)	Peg3-5 arm<br>Peg3-rev	CCCTCAGCAGAGCTGTTTCCTGCC<br>ACCCCATTCTCATCAGCTCCAGAG	2nd primer	≅700	mChr7: 6,669,691-6,668,412
KO2 (genotyping)	bac2082-F1<br>bac2375-R	ACAACCCGGAGTTTTAGCAGAC<br>AGGGGAGAACAGACTACAGA	1st primer	none	mChr7: 6,684,449-6,684,158
KO2 (genotyping)	bac2082-F2<br>bac6710-R	ACAACCCGGAGTTTTAGCAGAC<br>GGATGTAAGATGGAGGCACTGT	2nd primer	≅400	mChr7: 6,684,449-6,679,821
Zp3-cre (genotyping)	Zp3-cre-F<br>oIMR1085	TAGGAATCACGTGGAGTGTCT<br>GTGAAACAGCATTGCTGTCACTT	1st primer	≅500	mChr5: 136,455,787-unknown
**Primers used for 5'RACE and NGS**					
RACE gene-specific (KO2)	mEx2-R1	AGTCTTCCTCTTGCCAGTTGTC	1st primer	≅ RT	mChr7: 6,679,232
RACE nested (KO2)	mEx2-R2<br>mEx2-R3	TCCTCTTGCCAGTTGTCTCCAA<br>ATAGAAGATCAAGAAGGTAGGG	2nd primers	≅ identify	mChr7: 6,679,237
RACE gene-specific (DelKO)	mEx6-R2	CCAAAATGTGGTCTTGACATCACAG	1st primer	≅ RT	mChr7: 6,668,817
RACE nested (DelKO)	mEx6-R3<br>m12,4<br>U0-R	ATGTGGTCTTGACATCACAGGAAGA<br>TGTCACTGTTGGTGTCGTCT<br>CTGGCCATGTTGTATCATTGTA	2nd primers	≅ identify	mChr7: 6,668,822; 6,670,582; 6,699,528
RACE gene-specific (human)	hEx2-R1	TCCCTCTTCCTCTCGCCAGTCG	1st primer	≅ RT	hChr19: 56,836,049

RACE nested (human)	hEx2-R2<br>hEx2-R3	CTTCCTCTCGCCAGTCGTCTCC<br>CGGAAGATCAAGAAGGCAAAG	2nd primers	≅ identify	hChr19: 56,836,054; 56,836,084

RACE tailing primers	tail long<br>tail out	GGTTGTGAGCTCTTCTAGATCCCCCCCCCCCCNN<br>GGTTGTGAGCTCTTCTAGA	1st primer	≅ identify	5' end

Primers used for RT-PCR analysis

RT-PCR-mE1	Peg3-RT-1a<br>mEx6-R3	GGTTCAGTGTGGGTGCACTAGACT<br>ATGTGGTCTTGACATCACAGGAAGA	1st primer	≅500	mChr7: 6,683,015-6,668,822
RT-PCR-mU0	m-U0F<br>mEx6-R3	TACAATGATACAACATGGCCAG<br>ATGTGGTCTTGACATCACAGGAAGA	1st primer	≅600	mChr7: 6,699,528-6,668,822
RT-PCR-mU1	m-U1F<br>mEx6-R3	AGGCTTTCCATTCCCAGCATCT<br>ATGTGGTCTTGACATCACAGGAAGA	1st primer	≅800	mChr7: 6,703,897-6,668,822
RT-PCR-mU2	m-U2F<br>mEx6-R3	TGGCCACTTCAATTCTGGAAGG<br>ATGTGGTCTTGACATCACAGGAAGA	1st primer	≅800	mChr7: 6,709,557-6,668,822
RT-PCR-mU3	m-U3F<br>mEx6-R3	TCAGGTCACCAGGCTTCGACA<br>ATGTGGTCTTGACATCACAGGAAGA	1st primer	≅700	mChr7: 6,846,600-6,668,822
RT-PCR-mUsp29	mUsp29-1a<br>mUsp29-Ex2-R3	GAGGAGAGCAAGCAGGTAGATTAC<br>TGAAATGGGGAGTAGGGTGAAC	1st primer	≅180	mChr7: 6,731,145-6,737,586
RT-PCR-mβ-actin	β-actin-1a<br>β-actin-1b	GAGCACCCTGTGCTGCTCACCGA<br>CTCTTTGATGTCACGCACGATTTC	1st primer	344	mChr5:143,666,183-143,666,981
RT-PCR-hE1	hEx1-F1<br>hEx2-R2	GCAGAAGTCTGGGCAGCTGCGG<br>CTTCCTCTCGCCAGTCGTCTCC	1st primer	≅190	hChr19: 56,840,705-56,836,054
RT-PCR-hU1	hU1-F<br>hEx2-R2	AGCGTGTAGATGGCAAGCAGAGC<br>CTTCCTCTCGCCAGTCGTCTCC	1st primer	≅150	hChr19: 56,842,954-56,836,054
RT-PCR-hMimt1	hMimt1-Ex1-F<br>hMimt1-Ex2-R	GTCGAAATGGAGGAAACCCCAC<br>ACTGGATTTGGACTCTCCTGAC	1st primer	≅624	hChr19: 56,840,992-56,847,984

Primers used for DNA methylation analysis

Bis-E1	mPeg3-pro-bis-a.1<br>mPeg3-pro-bis-b	GTTTTTGTAGAGGATTTTGATAAGGAG<br>CACCCCAAACACCATCTAAACTCTACAAAC	1st primer	≅290	mChr7: 6,682,886-6,683,178

Bis-U1	bis-Peg3-RACEF1-F1<br>bis-Peg3-RACEF1-R1	GTTGGGAATGGAAAGTTTAAAGATAAA<br>AAAATCAAAACTACACCAAACATACAAC	1st primer	≅251	mChr7: 6,703,901-6,704,151
Bis-U2	ECR4-Bis-a<br>ECR4-Bis-b	ATTGGTTTATAGTTAGGGAAGGAAGTAGT<br>AAATCTCTCTAAAACATAATACTATTCTAT	1st primer	≅324	mChr7: 6,759,695-6,760,018
Bis-U3	bis-Peg3-RACEF5-F<br>bis-Peg3-RACEF5-R	GTAGGTAGATAATTTATTGGATAAAGAGTT<br>CTTTCTTTCTCTCTTTCTTTATACATATAT	1st primer	≅184	mChr7: 6,846,566-6,846,749
